# Maximum isometric torque at individually-adjusted joint angles exceeds eccentric and concentric torque in lower extremity joint actions

**DOI:** 10.1186/s13102-022-00401-9

**Published:** 2022-01-21

**Authors:** Andreas Stotz, Ebrahem Maghames, Joel Mason, Andreas Groll, Astrid Zech

**Affiliations:** 1 Department of Human Movement Science and Exercise Physiology, Institute of Sport Science, Friedrich Schiller University Jena, Seidelstraße 20, 07749 Jena, Germany; 2 Department of Statistics, TU Dortmund University, Vogelpothsweg 87, 44227 Dortmund, Germany

**Keywords:** Maximum muscle torque, Contraction type, Gravity, Joint torque angle

## Abstract

**Background:**

Previous research indicates the high relevance of optimal joint angles for individual isometric strength assessment. The objective was to compare lower limb peak isometric muscle strength abilities at the strongest joint angles with those of dynamic contractions in healthy young adults.

**Methods:**

Eighteen young male adults performed maximum concentric, isometric, and eccentric contractions of the ankle, knee, and hip flexors and extensors, and hip adductors and abductors in a randomized sequence on an isokinetic dynamometer (ISOMED 2000). Angular velocity was set at 60°/s. The peak of concentric contraction torque curves was used to define optimal joint angles best suited to generate maximum torque during isometric contractions. Maximum voluntary contraction torque of all contraction conditions was adjusted for limb weight and analyzed via a generalized linear mixed gamma regression model (GLMM).

**Results:**

The gamma GLMM revealed strongly significant effects for all three categorical covariates (contraction types, muscle group, and test order) ($p < 2 \times 10^{ - 16}$). Eccentric contraction increases the muscle torque ($\hat{\beta }_{k} = 0.147$) compared to concentric contraction, and isometric contraction even more ($\hat{\beta }_{k} = 0.258$). A moderate individual-specific variation was found (random effects standard deviation $\sigma_{b} = 0.093$).

**Conclusion:**

The results support the importance of optimal joint angles for isometric maximum strength assessment. When such conditions are given, isometric contractions can produce higher muscle torques than eccentric contractions in the lower body.

## Background

Muscular strength is the production of force against external resistance. It is therefore one of the most critical elements of human movement and a necessity to independently perform various tasks of daily living. Accordingly, strength assessments are regularly performed for clinical, rehabilitative, or sporting performance purposes [1–4], and a wide range of procedures exist for its assessment in different contexts [5–8]. Performance on these strength assessments is generally influenced by a range of testing parameters, including contraction speed, joint angle, testing apparatus, and the number of repetitions, and therefore comparability between studies and their various assessment methods is often limited [8–12].

One particularly influential aspect known to mediate not only the acute production of muscular torque, but also gains in muscular strength via training programs, is the type of muscle contraction [9, 13]. It is widely accepted that the highest torques are generated during eccentric muscle contractions, with previous studies demonstrating that isokinetic eccentric contractions produce torques 22–60% higher than concentric contractions [12, 14]. Isometric contractions typically demonstrate lower peak torques than eccentric contractions, but higher peak torques than concentric contractions [15–17]. However, the production of isometric peak torque is mediated by the length of the muscle, and the highest torque is produced at the muscle length where there is largest overlap between actin and myosin filaments [18, 19]. Consequently, isometric torque is highly variable based on muscle length, and joint angle must be carefully considered when testing for isometric strength [11, 20–23]. Accordingly, a closer proximity to the optimal joint angle for maximal expression of isometric torque may explain why some studies report no difference in maximal torque production between eccentric and isometric contractions of the knee extensors [24] and plantar flexors [25]. Other explanations such as the lack of familiarization of the participants with eccentric contractions inducing a deficit of muscle activation [26] (compared with isometric contractions) that is not found in trained individuals [24] might also be responsible for the lack of difference between eccentric and isometric peak torque.

Indeed, previous studies comparing torque between contraction types have been limited by the use of arbitrarily selected and pre-determined joint positions [17, 27–29], which potentially limits the peak torque reached during these contractions [30–32]. This leads to methodological constraints since the magnitude of peak joint torques is only reasonable to compare when the optimal joint torque angle is applied during isometric contractions [11, 23]. Although some recommendations for an optimal joint angle for maximum torque are available [33–37], deviations through individuals anthropometry exist, and therefore optimal joint angles unique to each individual should be obtained beforehand in order to produce a true maximal isometric torque measurement [15]. However, this has not been included in previous studies comparing torque differences between contraction types.

The aim of this study was therefore to compare maximum voluntary contraction performance during isometric contractions at individually-determined optimal joint angles with those of dynamic contractions across the main lower extremity joint actions. Novel insights about contraction type-dependent muscle torque could improve current knowledge about mechanisms of strength production.

## Methods

### Participants

Eighteen healthy males were recruited among the university’s student population via word of mouth for voluntary study participation (age 24.8 ± 1.9 years, height 181.6 ± 7.2 cm, mass 81.2 ± 8.5 kg). Participants were excluded from the study if they had an injury history in either the lower limb or torso within the previous six-month period. To ensure subjects would perform strength tests in a recovered state, they were instructed to not engage in strenuous physical activities within the two days before participation in the study. Participants signed written informed consent, and ethical approval was been obtained by the local Ethical Commission (protocol number: FSV 20/002) and all methods were performed in accordance with the relevant guidelines and regulations.

### Study design

Each participant attended a single session where the complete test was performed by an investigator with experience in isokinetic strength assessment.

To minimize the risk of bias through fatigue or warm-up effects, a counterbalanced crossover design was used where participants were randomly assigned to one of three groups. Group 1 started with concentric followed by isometric and eccentric muscle contractions, Group 2 also started with concentric contraction followed by eccentric and isometric muscle performance, and Group 3 started with the eccentric followed by concentric and ending with isometric muscle contractions.

### Assessment

Body height and weight were measured for each participant at the beginning. The dominant limb was determined by asking participants for their leg preference to kick a ball [38], and used for strength testing. Muscle strength for all three contraction types was measured with the Isomed 2000 system (Isomed 2000^®^, D&R Ferstl GmbH, Hemau, Germany).

The individual optimal joint angle for isometric assessment [23] of each muscle group was determined by using the peak of the torque curve during a maximum concentric contraction trial. In a preliminary test, joint angles derived from concentric tests produced higher peak strength values in subsequent isometric contractions than those derived from eccentric contractions. Therefore, we elected to use concentric contraction curves to define optimal isometric joint angles. To eliminate the influence of limb weights on muscle torques, the weight of the lifted respective extremity or part of it was annulled by the dynamometer [29].

After ensuring a full range of motion of the tested joint participants underwent a familiarisation and warm-up procedure lasting about five minutes. During the familiarization phase, participants tested the full range of motion of the single joint movement. This was especially important to be able to smoothly transition between flexion and extension. Also, subjects got familiar with the graphical feedback curves of the dynamometer. Afterwards, they kept warming up by practicing the movement with increasing but submaximal effort. Before testing subjects rested for 90 s after the warmup. While the order of contraction types was randomized, strength assessment was performed in a fixed order: (1) ankle plantarflexion and dorsiflexion, (2) knee extension and knee flexion, (3) hip extension and hip flexion, (4) hip abduction and hip adduction. Each muscle group and each contraction type were measured across two trials, with 90 s rest between each. The assessments were performed with an angular velocity of 60° per second. Isometric muscle contractions were performed with a duration of 3 s. The higher peak torque reached across two trials was used for analysis.

#### Ankle plantarflexion and dorsiflexion

This assessment was performed in a seated position. Firstly, the subject’s foot was placed on the platform of the adapter that is designed for strength assessment of ankle plantarflexion and dorsiflexion. The length of the adapter was adjusted to the subject’s lower leg length. Next, the pivot point of the ankle joint (malleolus lateralis) and the dynamometer were synchronized to each other. After that, the seat position was adjusted to the femur length and anthropometrics of the pelvis so that the knee joint bent at 90° while the foot was still positioned on the adapter. With this arrangement, the hip of the tested side was flexed at around 45° while the other side was at 90°. An additional support pole was set on the back of the tested leg to prevent the subject from pushing via leg extension on to foot platform and therefore influence plantarflexion strength results. Lastly, to prevent evasion movements the subject’s foot, ankle, and femur were fixed with straps, the pelvis was strapped with a belt, shoulders were restrained with shoulder pads. The tested range of motion ranged from − 20° of dorsiflexion to 35° of plantar flexion. The 0° position for the ankle joint was defined as a neutral position where the joint is neither plantar nor dorsiflexed.

#### Knee extension and knee flexion

This test was also performed seated. At first, the seat and adapter length were adjusted to the femur and shank length, respectively. To transfer the strength of the thigh muscles to the pad, the adapter was fixed to the distal part of the shank slightly above the malleoli. Next, the pivot point of the knee joint (center of the knee joint) and the device were synchronized to each other. The backrest and seat were slightly inclined so that the hip angle was around 90°. The initial angle of the knee was 90°. Lastly, to prevent evasion movements the subject’s femur was fixed with straps, the pelvis was strapped with a belt, shoulders were restrained with shoulder pads.

The tested range of motion ranged from 5° to 90° of knee flexion.

#### Hip abduction and hip adduction

The assessment of hip abduction and hip adduction strength was performed in a laying position with hips and knee joints being in a neutral position at 0°. Participants laid on the right side to test the left hip muscles and vice versa. The pivot point of the frontal plane of the hip joint (femoral head) and the device was synchronized to each other and was adjusted for pelvis height. The adapter length of the testing device was adjusted to the femur length and was attached to the distal part of the femur. The lower hip and leg (not tested side) were slightly bent and the leg was fixed to the bench at the femur so that the leg and body were grounded and an elevation of the body (in case the participant was strong enough) was hindered. Additionally, a belt was attached around the pelvis to further prevent evasion movement. The tested range of motion ranged from 0° to 60° of hip abduction.

#### Hip extension and hip flexion

This strength evaluation was performed with the subject laying on the back with hips and knee joints being in a neutral position at 0°. The pivot point of the sagittal plane of the hip joint (trochanter major) and the device was synchronized to each other. The adapter length of the testing device was adjusted to the femur length and was attached to the distal part of the femur. A belt was attached to the pelvis to avoid its elevation during hip extension. Furthermore, the shoulders were restrained with pads to prevent the participant from sliding off the bench and to provide opposition to express torque against the adapter. The non-testing leg was passively laying on the bench was not allowed to facilitate the movement of the tested leg. The tested range of motion ranged from 10° to 100° of hip flexion.

### Statistical analysis

Statistical analyses were performed with JASP (JASP Team 2020, version 0.12.2) and R (R Core Team, 2020; version 4.0.3). Descriptive data are reported as mean and standard deviation. Regression analyses via a (Gaussian) linear mixed model and a gamma generalized linear mixed model (GLMM) with log-link were performed via the R package lme4 [39] to investigate the effects of different contraction and flexion types as well as of the group assignment on maximum muscle torque. Individual-specific heterogeneity was accounted for by the inclusion of corresponding random intercepts. The models were compared via the Akaike information criterion (AIC; [40]) and by the investigation of residual plots, and the gamma GLMM turned out to be clearly superior with respect to both aspects.

## Results

**Fig. 1:**
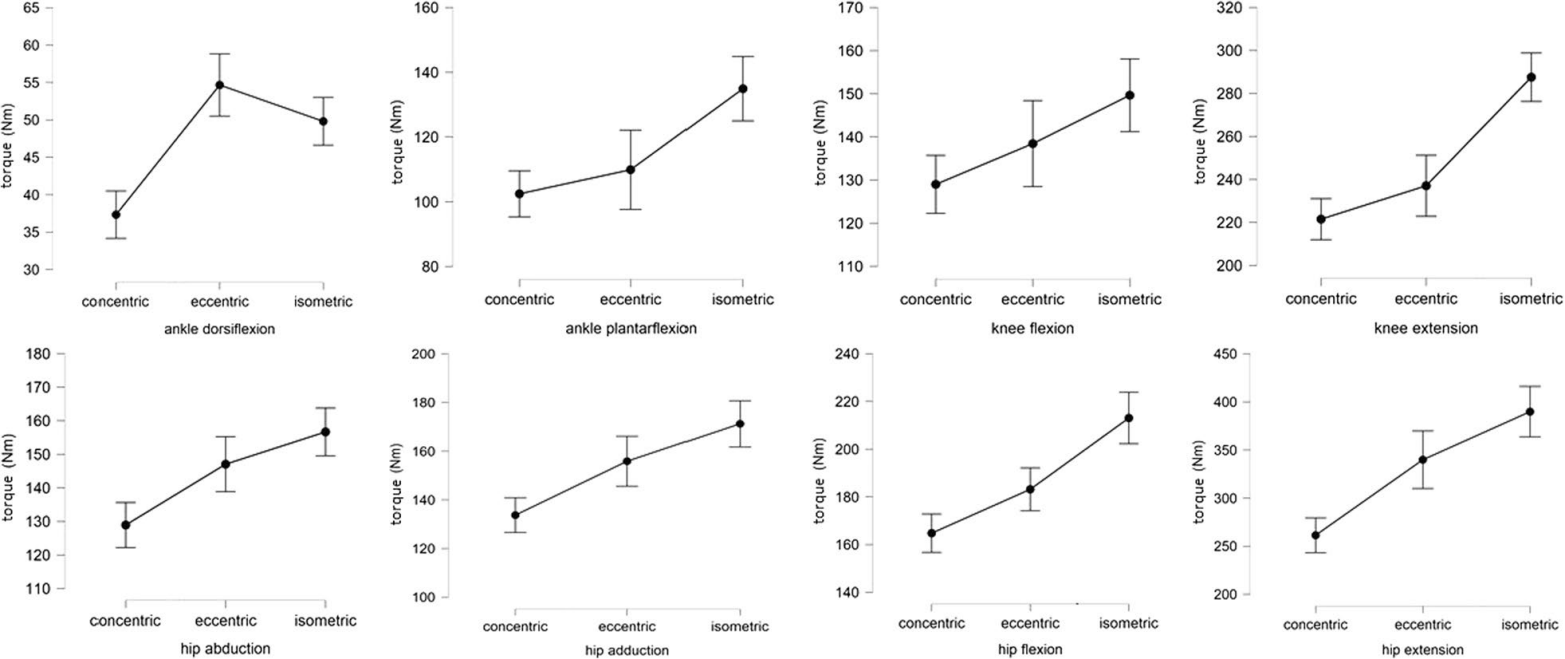
Maximum muscle torque and standard deviation for each muscle group and contraction type

Figure 1 presents maximum muscles torque and standard deviation for each muscle group and contraction type. Mean and standard deviations of individually determined optimal joint angles used for isometric assessments are shown in Table 1.

**Table 1:** Mean and standard deviation of maximum torque angles during maximum concentric contractions (PF = plantar flexion, KF = knee flexion, HAb = hip abduction, HF = Hip flexion, zero-degree-position is defined as neutral upright standing according to [41])

Muscle groups	Angle at peak MVC
Ankle plantarflexors	7° ± 4° PF and 90° KF
Ankle dorsiflexors	20° ± 4° PF and 90° KF
Knee flexors	37° ± 14° KF and 90° HF
Knee extensors	65° ± 5° KF and 90° HF
Hip abductors	13° ± 5° HAb
Hip adductors	25° ± 12° HAb
Hip flexors	25° ± 6° HF
Hip extensors	75° ± 11° HF

The gamma GLMM revealed strongly significant effects for all three categorical covariates ($p < 2 \times 10^{ - 16}$), see Table 2, where concentric contraction, plantar flexion and test order group 1 where chosen as reference categories for the categorical covariates. Due to the used parametrization of the gamma density in combination with the log-link, precise interpretation of the effect sizes is difficult, but positive effects increase the muscle torque, while negative effects decrease it. For example, excentric contraction increases the muscle torque ($\hat{\beta }_{k} = 0.147$) compared to concentric contraction, and isometric contraction even more ($\hat{\beta }_{k} = 0.258$). Accordingly, all muscles groups ($\hat{\beta }_{k} \in \left[ {0.191;1.045} \right]$) besides dorsi flexion ($\hat{\beta }_{k} = - 0.882)$ produced higher torques. Lastly, results show that subjects with the test order 1 were stronger compared to individuals in test orders 2 ($\hat{\beta }_{k} = - 0.212)$ and 3 ($\hat{\beta }_{k} = - 0.207)$. A moderate individual-specific variation was found (random effects standard deviation $\sigma_{b} = 0.093$) (Table 1).

**Table 2:** Results of a gamma GLMM include individual-specific random intercepts; the global intercept represents the reference level of the three categorical predictors (i.e., concentric contraction, plantar flexion, test order 1), *p* value *** < 0.001

	Estimate	Std. error	t Value	Pr ( >|z|)	
(Intercept)	4.722	0.009	527.67	$< 2 \times 10^{ - 16}$	***
Eccentric contraction	0.147	0.008	17.50	$< 2 \times 10^{ - 16}$	***
Isometric contraction	0.258	0.009	29.34	$< 2 \times 10^{ - 16}$	***
Dorsi flexion	− 0.882	0.009	− 94.52	$< 2 \times 10^{ - 16}$	***
Knee flexion	0.191	0.009	20.64	$< 2 \times 10^{ - 16}$	***
Knee extension	0.778	0.030	25.60	$< 2 \times 10^{ - 16}$	***
Hip abduction	0.235	0.009	26.90	$< 2 \times 10^{ - 16}$	***
Hip adduction	0.297	0.009	31.97	$< 2 \times 10^{ - 16}$	***
Hip flexion	0.495	0.009	54.58	$< 2 \times 10^{ - 16}$	***
Hip extension	1.045	0.009	121.89	$< 2 \times 10^{ - 16}$	***
Test order 2	− 0.212	0.009	− 23.57	$< 2 \times 10^{ - 16}$	***
Test order 3	− 0.207	0.009	− 22.93	$< 2 \times 10^{ - 16}$	***

## Discussion

This is the first study to directly compare peak torque between maximal voluntary concentric, eccentric and individualized isometric contractions of multiple joints. Our findings indicate that when individualized joint angles are applied, isometric contractions produced larger torques than eccentric in reference to concentric contractions. Further, concentric contractions always produced less torque than isometric contractions, and typically less than eccentric contractions.

The higher isometric peak torque compared to eccentric peak torque contradicts the current understanding of the hierarchy of contraction types for maximum voluntary torque production. This understanding is based on numerous studies showing the outstanding role of eccentric contractions compared to concentric and isometric contractions [15–17, 42, 43]. Differences between the results of our study and the existing literature may be driven by the methodological approach, primarily our use of individualized and optimized isometric joint angles as opposed to an arbitrarily determined joint angle that is shared between all participants [17, 27–29].

Indeed, the joint angles used closely resemble those used in other isometric strength assessment studies that were not designed to compare torque during different contraction types, including for hip flexion and extension [44], hip adduction and abduction [36], and knee flexion and extension [30, 33, 37]. By individually optimizing the joint angle to the point where the torque was highest, we maximized the torque-length relationship of the muscle by theoretically locating the point with the most filament overlap [18, 19, 45]. This may help to explain why we saw higher isometric torques than other studies because they used different joint angles and therefore different muscle lengths which did not maximize this torque-length relationship. However, the sliding filament theory was described at the microlevel of a sarcomere, and therefore applying the principles to a whole muscle is a complex process [45]. Factors such as transmission efficiency [46], muscle architecture [47] including geometric arrangement of muscle fibers [48] and configuration of the lever system of the joint [49] influence contractile properties of the muscle, each of which may uniquely influence the isometric torque an individual can produce at a given angle [37].

Additional underlying physiological mechanisms beyond the overlap of filaments may also explain why the joint angle-optimized isometric contractions demonstrated higher torques than other contraction types. For example, twitch interpolation studies indicate that during maximal anisometric contractions, voluntary activation of the quadriceps femoris is significantly lower than during maximal isometric contractions of the same muscle [50]. This may be explained by neuromuscular inhibition levels unique to anisometric contractions, at both a spinal and cortical level [51]. Further, antagonist muscle co-activation is known to be specific to the joint being tested and potentially specific to the mode of contraction [52], which may explain differential effects between different muscle groups and actions in the current study. Finally, muscle activation during isometric contraction fluctuates according to joint angles not just for the agonist muscle [30] but synergist muscles too [53, 54], and so individually adjusting the angle of assessment may have facilitated greater synergist muscle contributions, driving the achievement of higher peak torques. Another plausible explanation for our findings is that without a true familiarization period, the methods of testing may have underestimated maximal eccentric torque. Due to the commonly reported difficulties in achieving full activation of a muscle by voluntary command during eccentric contractions [55], it is possible that deficits in motor control may have influenced maximal eccentric torque magnitude.

Besides the novel findings that lower body muscles produced higher isometric than eccentric torques, we found that concentric contractions produced the lowest peak torque values, which is supported by a considerable volume of existing literature for hip extension [12], hip abduction [28], knee extension, and flexion [17, 27, 29, 50, 56, 57], ankle plantarflexion [17, 25] and ankle dorsiflexion [17, 58–60].

Beyond the use of individualized isometric joint angle, other elements of experimental design potentially influenced divergent results between studies. This includes the use of an isokinetic angular velocity of 60°/s while [28] used 13°/s and [15] used 36°/s. In our study, only young male participants were included while [17, 27], also included female participants, and [29] measured female subjects exclusively. We adjusted all measurements for limb weight while all but two other studies [28, 29] did not. Lastly, it should be noted that early reports simply compared absolute torque values between contraction modes, therefore using insufficient analysis to draw reasonable statistical conclusions regarding the hierarchy of contraction modes [17, 28].

## Limitations

The current study has some limitations that must be considered alongside the results. Because we used a GLMM with dummy encoding for the three categorical predictors, all their effect sizes ($p < 2 \times 10^{ - 16}$) are directed towards the chosen reference categories (concentric contraction, plantar flexion and test order group 1). Hence, we can only interpret the effect of one covariate when the other two remain constant. This complicates the comparison between contraction types within one muscle group (comparison of more than one covariates). A particular example is that dorsiflexion eccentric torque was higher than isometric and eccentric torque but this information is not detectable in the model. Moreover, as a gamma GLMM with log-link clearly outperformed a Gaussian linear mixed model, this also complicates the precise interpretation of the regression coefficient estimates. Only one angular velocity of 60°/s was used for the comparison of maximum torque between contractions types. Previous studies have indicated that maximum isokinetic torque generally gets lower with higher angle velocities [23, 29], and also that this force–velocity relationship may be contraction-type dependent [61, 62]. It has to be noted additionally, that previous research has shown that the highest torque angle shifts with increasing angular velocity [63]. In this study, we used the optimum torque angle for isometric measurement derived from the torque curves from the concentric contractions. Although we found the highest torque angle for every participant, an even better torque angle might exist with the assessment of concentric torque with a velocity below 60°/s. Still, this approach is novel and previous works measured isometric torques through an array of angles while potentially not always finding the optimal one. Another limitation of this study is the absence of a true familiarization period of the participants with eccentric contractions. This might trigger a repeated-bout effect and allow for a limited so-called descending command inhibition [64]. Also, no measure of voluntary activation such as electromyography and voluntary activation via twitch interpolation techniques was provided to judge possible activation deficit during eccentric contractions. An alternative to a separated familiarization session could have been the usage of isometric pre-tension contraction before the onset of each eccentric contraction to maximize voluntary activation [26]. Lastly, our study was performed with young healthy males, and therefore the results may not be generally transferable to other populations like seniors or patients with specific pathological conditions.

Future studies are therefore encouraged to examine maximum torque of the three contraction types in other populations [65], examine strength relationships in both genders and also test other upper-body muscle groups. With the loss of strength being one of the most important factors for functional decline in the elderly [66], new insight about contraction-specific torque could help developing intervention programs for the prevention of falls and sarcopenia.

## Conclusion

Maximum torques of the lower body differ significantly between contraction types, and this may be specific to the joint action, muscle groups used and the context of the testing. While further studies including more mechanistic insight are encouraged, our current findings suggest that at individually adjusted joint angles, maximum isometric torque can exceed eccentric and concentric torque in lower extremity joint actions. We, therefore, recommend that individually-optimized joint angles should be obtained prior to comparing maximum torques between isometric and dynamic contractions. Further, isometric strength assessments before and after training should maintain identical joint angle between tests due to the significant influence on the torque generated by a muscle group. Combined, our findings emphasise the need for a suitably controlled testing environment, and add to existing knowledge about mechanisms of strength production which may be useful not only for the assessment of muscular strength, but also the development of contraction type-specific intervention programs in a range of populations and contexts.

## Data Availability

All data generated or analysed during this study are included in this published article.
